# PPARG: Gene Expression Regulation and Next-Generation Sequencing for Unsolved Issues

**DOI:** 10.1155/2010/409168

**Published:** 2010-09-08

**Authors:** Valerio Costa, Maria Assunta Gallo, Francesca Letizia, Marianna Aprile, Amelia Casamassimi, Alfredo Ciccodicola

**Affiliations:** ^1^Institute of Genetics and Biophysics “Adriano Buzzati-Traverso” (IGB), CNR, 80131 Naples, Italy; ^2^“Centro Diagnostico San Ciro” (CDS), 80055 Portici (NA), Italy; ^3^Department of General Pathology, 1st School of Medicine, Second University of Naples, 80138 Naples, Italy

## Abstract

Peroxisome proliferator-activated receptor gamma (PPAR*γ*) is one of the most extensively studied ligand-inducible transcription factors (TFs), able to modulate its transcriptional activity through conformational changes. It is of particular interest because of its pleiotropic functions: it plays a crucial role in the expression of key genes involved in adipogenesis, lipid and glucid metabolism, atherosclerosis, inflammation, and cancer. Its protein isoforms, the wide number of PPAR*γ* target genes, ligands, and coregulators contribute to determine the complexity of its function. In addition, the presence of genetic variants is likely to affect expression levels of target genes although the impact of *PPARG* gene variations on the expression of target genes is not fully understood. The introduction of massively parallel sequencing platforms—in the Next Generation Sequencing (NGS) era—has revolutionized the way of investigating the genetic causes of inherited diseases. In this context, DNA-Seq for identifying—within both coding and regulatory regions of *PPARG* gene—novel nucleotide variations and haplotypes associated to human diseases, ChIP-Seq for defining a PPAR*γ* binding map, and RNA-Seq for unraveling the wide and intricate gene pathways regulated by PPARG, represent incredible steps toward the understanding of PPAR*γ* in health and disease.

## 1. Introduction

Gene transcription requires an elaborate network of intra- and extracellular signals, such as hormones, xenobiotics, micro- and macronutrients (lipid metabolites, vitamins, ions, etc.) and drugs, that converge to the nucleus following different pathways, resulting in the expression of each gene in each tissue. It is a current assumption that transcription is mostly shaped by environmental factors, acting both via direct and indirect mechanisms. Translating exogenous and endogenous signals which affect gene transcription, into a cellular physiological response requires the coordinated action and the fine tuning of transcription factors (TFs) acting at DNA level, including those belonging to the nuclear receptor (NR) superfamily [1, 2]. 

The human NR superfamily comprises 48 ligand-inducible transcription factors that respond to a variety of *stimuli* and are able to modulate their transcriptional activity through conformational changes [3]. The most extensively studied members of this TF superfamily are the Peroxisome proliferator-activated receptors (PPARs, also known as nuclear receptor family 1C, NR1C). Crystallographic studies have shown that all NRs superfamily members, and among them PPARs, share common structural features which include a poorly conserved N-terminal A/B domain (with a potential transactivation domain AF-1), a highly conserved DNA binding domain (DBD), with two zinc finger motifs, a C-terminal region containing the ligand-binding domain (LBD) and confer the ligand-dependent transactivation function (AF-2), and a length-variable hinge region between the DBD and LBD [4].

PPARs function as heterodimers with retinoid X receptor (RXR, NR2B), and their TF activity is regulated by the binding of ligands, the interactions with coregulators (both activator and repressor proteins), and DNA-binding sites [5]. 

In a basal state, the PPARs-RXR complex is bound to corepressors and is transcriptionally inactive. The binding of endogenous or synthetic ligands to the AF-2 domain promotes a conformational change, which results in the release of corepressors, allowing the recruitment of*—*and the interaction with*—*coactivators [6]. These proteins either possess or recruit proteins with, histone acetyltransferase (HAT) activity allowing the RNA polymerase II complex to bind and initiate transcription of target genes [4, 7].

PPAR genes are expressed in different organs, such as reproductive and major insulin target organs—liver, white and brown adipose tissue (WAT and BAT, resp.), and skeletal muscle—cardiac tissue, and others [8]. They have been implicated in different biological pathways ranging from lipid and glucose homeostasis and insulin sensitization, to control of cell proliferation/differentiation, tissue injury and wound repair, inflammation, and immunity [9]. 

PPARs occur in three different isotypes, termed *α* (NR1C1), *β*/*δ* (NR1C2), and *γ* (NR1C3), encoded by three separate genes, localized on human chromosome 22q12-q13.1 [10], 6p21.2-p21.1 [11], and 3p25.2 [12], respectively, and expressed in a tissue-specific manner. All three isotypes are able to bind, with different affinities, the same consensus response element on DNA, named peroxisome proliferator response element (PPRE) [13]. Despite their substantial homology and evidence of shared transcriptional targets, the physiological functions of each PPAR are unique. 

PPAR*γ*, the best studied member of the PPAR family, is induced during the differentiation of preadipocytes into adipocytes and is expressed most abundantly in WAT and BAT [14]. PPARs have a great relevance in the human physiology, but they are also involved in the etiology of many human diseases, and, in this contest, PPAR*γ* is of particular interest because of its pleiotropic functions: it plays a dominant role in the control of the expression of a plethora of genes related to a wide spectrum of physiological processes, such as adipose cell differentiation, metabolism, atherosclerosis, inflammation, and cancer. 

 Alternative promoters usage and mRNA splicing give rise to at least seven PPAR*γ* isoforms: the 5′ end of the mRNA consists of alternately spliced exons A1, A2, B, C, and D in various combinations. Each splice variant differs only in the 5′-UTR: the exons at the 5′ end account for little or none of the final translated PPAR*γ* protein [15, 16]. In particular, the well-studied PPAR*γ*1 and PPAR*γ*2 have distinct N-terminal portions, differing by the presence of extra 28 (mouse) -30 (human) amino acids for PPAR*γ*2 isoform [17, 18]. PPAR*γ*1, whose expression can be regulated by multiple promoters (*γ*1, *γ*3, and *γ*4), is expressed in all PPAR*γ*-expressing tissues and cells whereas PPAR*γ*2 is almost exclusively found in adipose tissue [19, 20], where it exerts a pronounced adipogenic activity.

Two *PPARG* gene 3′ splice variants*—*lacking almost the entire LBD*—* *γ*ORF4 and PPAR*γ*1_tr _, have been identified as dominant negative *versus* PPAR*γ* wild type [21, 22]; hence they are not able to promote the transactivation of *PPARG* target genes. 

The significant number of PPAR*γ* isoforms, as well as for other NRs, strongly suggests that splicing plays an important role in the nuclear receptor functioning. Moreover, the large number of PPAR*γ* target genes, ligands, and coregulators (both coactivators and corepressors) confers additional complexity to PPAR*γ* function. In addition, alterations in the PPAR*γ* trans-activating ability have to be analyzed in the light of environmental factors, genetic background, and the interactions among them [23]. 

This paper summarizes the transcriptional regulation exerted by PPAR*γ* on key target genes and the effects of the most frequent *PPARG *gene nucleotide variations on its function, also approaching to the next generation sequencing (NGS) technologies that will allow an unprecedented level of accuracy and completeness to the study of PPAR*γ* and other transcription factors. Indeed, this paper describes in detail how these novel technologies will allow to identify novel genetic variants and polymorphisms (SNPs) in PPARG gene, to draw high-resolution binding map of PPAR*γ* across the genome, and to understand the transcriptional regulation of PPAR*γ*-modulated genes.

## 2. PPARG and Gene Expression Regulation (Target Genes)

PPAR*γ* controls several arrays of biological processes by modulating the expression of specific target genes mainly through a ligand-dependent mechanism [24]. PPAR*γ* ligands include a surprisingly diverse set of natural ligands [25] such as prostaglandin PGJ2, linolenic, eicosapentaenoic, docosahexaenoic, and arachidonic acids, and synthetic ligands, such as the thiazolidinediones (TZDs), L-tyrosine-based compounds, several nonsteroidal anti-inflammatory drugs, and a variety of new chemical classes.

The PPARs, and PPAR*γ* among these, like many nonsteroid members of the NR family, function as obligate heterodimers with RXRs [26]. The heterodimers are able to bind PPRE, consisting of direct repeats of the canonical AGGTCA half-site separated by one base pair (DR1) together with the upstream specificity element AAACT [13, 27]. Typically, RXRs do not function alone but rather serve as master regulators of several crucial regulatory pathways, in combination to different NRs' partners. 

**Figure 1:**
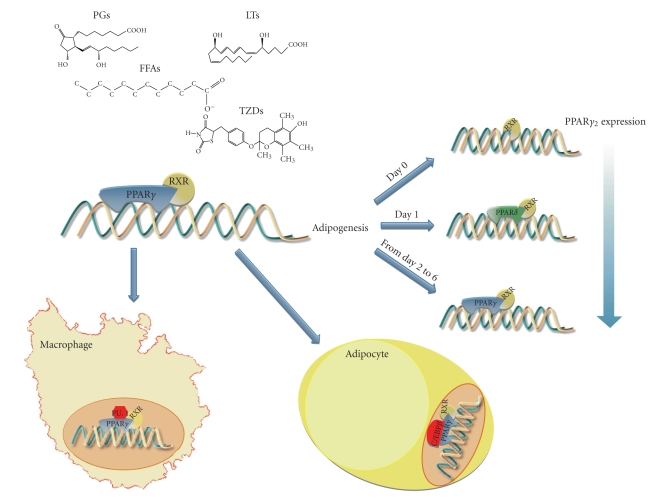
Novel insight into PPARG world trough new approaches. In the presence of ligands (upper), PPAR*γ* binds to its PPRE as heterodimer with RXR to activate or repress target genes' expression. The figure summarizes novel molecular mechanisms of PPAR*γ* obtained through ChIP-seq. PPAR*γ*- and RXR-binding sites detected by ChIP-seq reveal different spatial and temporal activation of distinct metabolic pathways and changes in RXR dimer composition during adipogenesis (right panel, study from [28]). *P* *P* *A* *R* *γ* *in Adipocytes and Macrophages:* tissue-specific regulatory regions employ cell-type-specific coregulators, C/EBP*β* in adipocytes and PU.1 in macrophages (lower panel; ChIP-Seq study from [31]).

Recently this issue has been better elucidated through the use of standard chromatin immunoprecipitation (ChIP) coupled with massive sequencing on NGS platform (described more in detail in Section 4.2 entitled “*Transcription Factors and ChIP-Seq*”) [28]. In this study the authors profiled PPAR*γ*- and RXR-binding sites throughout adipogenic differentiation (Figure 1). They identified differential spatial and temporal recruitment of PPARs and RXR to target sites during adipogenesis; in particular, at the onset of differentiation the DNA occupancy by RXR alone was detected. Interestingly, immediately afterwards, many of these sites become occupied by RXR and PPAR*δ*, lowly expressed into adipocytes. Moreover, through the early days of differentiation, they observed a different temporal and compositional pattern of occupancy with a switch between PPAR*δ* and PPAR*γ*, which becomes the main RXR partner throughout the adipogenesis, coinciding with a significant increase in both PPAR*γ*1 and PPAR*γ*2 expression [28–30]. The binding of RXR alone*—*in the early stage of differentiation*—*on the target sites later bound by PPAR*γ*:RXR complex has been hypothesized to serve as a signature needed for subsequent PPAR*γ*-dependent binding and/or activation of transcription for target genes [28]. 

The modulation of transcription depends on the recruitment of cofactors able to remodel the chromatin structure making it more accessible to the basal transcription machinery recruitment and assembly at the core promoter of target genes [32, 33]. Indeed, it has been widely assumed that chromatin accessibility to the transcriptional machinery, through histone modifications (acetylation, methylation, phosphorylation, ubiquitylation, sumoylation, deimination, ADP ribosylation, and proline isomerization) represents, a very relevant process into gene expression regulation [34–37]. In light of this, the different temporal*—*and compositional pattern*—*of occupancy on these binding sites, observed by Nielsen and colleagues (2008) [28] is likely to be required for the chromatin remodeling to such *loci*, rendering these regions accessible for PPAR*γ*:RXR binding and the subsequent transactivation of target genes. 

Although PPAR*γ*:RXR heterodimer controls the expression of many inducible genes, transcription is regulated both globally and locally by different factors. Determining which cell-specific coactivators/corepressors are recruited by PPAR*γ* in different cell types, and how these may contribute to chromatin modifications and differential gene expression, represents a crucial issue for fulfilling our gap towards the understanding of PPAR*γ* biology and function.

The currently assumed dogma, mostly referred to all TFs, is that the cell-type-specific trans-activating ability is due to the cooperative binding to other cell-type-selective factors, which specifically “drive” the TF to its target genes.

However, although it is well known that PPAR*γ* is able to modulate target genes' expression in some cell types but not others, the molecular mechanisms underlying its ability are not yet well elucidated. Differential binding of PPAR*γ* to the PPRE of target genes or its differential activity at DNA level (i.e., in chromatin remodelling) has been claimed as the putative mechanisms accounting for the cell-type specificity of its action [38].

A very recently published work of Lefterova and colleagues (2010) [31] has provided novel intriguing insight into the molecular basis of cell-type-specific gene expression in primary mouse adipocytes and macrophages. The authors, by using ChIP-Seq (see Section 4.2), identified the molecular signatures of PPAR*γ* binding, disclosing distinct macrophage- and adipose-specific PPAR*γ*-binding sites overall the genome. Moreover, they shed light on the cell-specific expression of PPAR*γ* target genes, demonstrating the tight and well-regulated cooperation of PPAR*γ* and other crucial cell-type-specific proteins (PU.1 and C/EBPb, nearby macrophage- and adipocyte-specific target genes, resp.) (see Figure 1). “PPAR*γ* dances with different partners” [38], and all the biological processes PPAR*γ*-modulated can be thus attributed to a differential recruitment of coactivators and corepressors functioning as scaffolds for chromatin remodelling enzymes. 

The coactivators of PPAR*γ* include well-established cofactors such as p300/CBP, p160, and PGC-1 (PPAR*γ* coactivator-1), as well as TRAP220 (thyroid hormone receptor-associated protein 220 or PBP, PPAR*γ*-binding protein) [39, 40], ARA70 (Androgen Receptor-Associated protein) [41], and PRIP (PPAR*γ*-interacting protein, ASC-2/RAP250 /TRBP/NRC) [42]. 

In the absence of ligand, PPAR*γ* recruits corepressors such as silencing mediator for retinoic and thyroid hormone receptors (SMRT) and the nuclear receptor corepressor (N-Cor), which bind repressive enzymes such as histone deacetylase enzymes (HDAC), and particularly HDAC3 [43] or the histone methyl transferase (HMT) SUV39H1, which specifically methylates histone H3 at lysine 9 (H3K9) [44]. RIP140 (receptor-interacting protein) may also be a component in the corepressor complex [45, 46]. The ability of PPAR*γ* to repress transcriptional responses to diverse signaling pathways is an essential aspect of its biological activities, but mechanisms determining the specificity and functional consequences of the process known as transrepression remain poorly understood. However, PPAR*γ* can also influence gene expression independently of its binding to the PPRE. Indeed, PPAR*γ*-dependent repression of inflammatory gene expression occurs through interference with the action of NF-kB via transrepression [47]. Moreover, the activity of other transcription factors, for example, AP-1 and STAT-1, can be inhibited by PPAR*γ* via direct interaction or by competition for limiting supplies of coactivators [48]. 

PPAR*γ* transactivation ability is induced by ligand-dependent and independent mechanisms. The AF-1 domain of PPAR*γ* is the ligand-independent activation domain that regulates the specificity of PPAR*γ* transcriptional activity during adipogenesis [49]. The presence of an extra 30 aminoacids in the AF-1 domain of PPAR*γ*2 isoform that makes it a better transcriptional activator than PPAR*γ*1 [50]. Indeed, it was shown that PPAR*γ*2 is about 10 times more active than PPAR*γ*1 in ligand-independent transcriptional activation, through this domain [50, 51]. Thus, PPAR*γ*1 and PPAR*γ*2 may have different functions, with PPAR*γ*1 being used when the ligand is abundant whereas PPAR*γ*2 would be crucial under conditions of low ligand concentration, such as it might occur in early adipocyte differentiation [51]. However, the ligand-independent transactivation through the AF-1 domain, common to PPARs, is poorly understood and beyond the scope of this paper.

### 2.1. PPARG-Modulated Pathways: Obesity and Inflammation

The biological activities of PPAR*γ* are very wide but it is generally acknowledged as a transcriptional regulator of lipid and glucose metabolism, since it is highly expressed in adipocytes and controls the expression of several adipocyte-specific genes involved in lipid synthesis and storage, insulin signalling, and adipokine production [52, 53]. 

PPAR*γ* ^−/−^ mice models, with selective ko in three metabolic tissues (adipose tissue, skeletal muscle, and liver), have revealed that PPAR*γ* is a master regulator of adipogenesis; PPAR*γ* deficiency and/or partial disruption in any of these tissue severely affects whole body lipid homeostasis, altering insulin sensitivity. The essential role of PPAR*γ* in adipogenesis was revealed by inactivation of both PPAR*γ*1 and PPAR*γ*2 in the adipose tissue [54, 55].

PPAR*γ*2 depletion was shown to dramatically diminish adipose tissue (WAT) mass*—*due to a strongly reduced adipocyte differentiation observed also *in vitro—*providing protection against high-fat-diet induced weight gain and to determine an impairment of insulin sensitivity [56]. In this context, a common aminoacid polymorphism (Pro12Ala) in PPAR*γ*2 (described in detail in the next section) has been associated with type 2 diabetes and has been suggested to induce a modest impairment of transcriptional activation due to decreased DNA-binding affinity [57].

Conflicting results have been reported by Medina-Gomez and colleagues (2005) [58]. Although they observed a clear *in vitro *defect in fat cell differentiation, they demonstrated that PPAR*γ*2-depletion is directly linked to insulin resistance, without alteration of *in vivo *adiposity, even in presence of a high-fat diet. The possible explanation of a residual presence of fat depots in these ko mice strongly suggested that PPAR*γ*1 was able to initiate, at least in part, adipocyte differentiation. In addition, it has been shown a global deregulation in the repartitioning of lipids in these mice models. A complex cross-talk between these metabolically active tissues (liver, adipose tissue, and muscle) appears to be essential for energy balance.

Other studies have demonstrated that mouse models of heterozygous PPAR*γ* (PPAR*γ* ^−/+^), with a decreased *PPARG* gene expression, show improved insulin sensitivity compared to wt mice [59, 60] although the reduced *PPARG *gene expression was associated with decreased metabolic rate and physical activity [61]. Reduction of *PPARG* gene expression in the PPARG^−/+^ mouse model is associated with a mild decrease in PPAR*γ* protein levels [62], suggesting that modulation of PPAR*γ* protein levels, rather than mRNA itself, may play a role in determining PPAR*γ* activity in adipocytes. Indeed, regulation of PPAR*γ* protein translation is expected to be tightly regulated. Althoguh a moderate decrease of PPAR*γ* protein may protect against high-fat diet-induced insulin resistance, its complete lack in adipocytes is deleterious to lipid and glucose metabolism as well as insulin sensitivity in the presence of a high fat diet, as shown in most, but not all, studies of adipose-specific PPAR*γ* knockout mouse models [63].

A considerable role for PPAR*γ* in macrophage lipid metabolism has been also clearly demonstrated [64]. The involvement of PPAR*γ* in regulating lipid metabolism in macrophages was initially suggested by the discovery of CD36, member of scavenger receptor family that mediates uptake of oxidized LDL, as a PPAR*γ* target gene in macrophages [65].

PPAR*γ* has a similar function in macrophages and adipocytes as it modulates lipid homeostasis in both cell types via regulation of genes including *LPL* (lipoprotein lipase), *ACAT* (acetyl coenzyme A acetyltransferase) and *PLA *(phospholipase A) genes, and the levels of *FFAs* (free fatty acids), *PGs* (prostaglandins), and *LTs* (leukotriens). PPAR*γ*-deficient mice have provided clues to an antiatherogenic role of PPAR*γ* since these mice showed a significantly impaired lipid homeostasis in the arterial wall and enhanced atherosclerosis development [66, 67]. The molecular mechanisms underlying the antiatherogenic properties of PPAR*γ* involve stimulation of cholesterol efflux from macrophages into the plasma and inhibition of monocyte recruitment into the developing atherosclerotic lesion [67]. Interestingly, macrophage-specific ablation of PPAR*γ* resulted in high rates of insulin resistance suggesting that macrophage PPAR*γ* may exert a protective role in obesity [68]. 

Indeed, it is becoming always more evident a functional link between macrophage activity, inflammation, adipose tissue, and type 2 diabetes mellitus (T2DM) [69, 70].

In a physiological state, macrophages residing in fat mass are responsible for keeping in the adipose tissue an anti-inflammatory environment, conferring an adequate degree of insulin sensitivity. In pathological conditions, such as obesity, adipose tissue is continually under metabolic stress, leading to the constitutive activation of stress and inflammatory pathways, resulting in macrophage accumulation within the adipose tissue. Proinflammatory macrophages infiltrate adipose tissue, exacerbating local inflammation and giving rise to insulin resistance [31]. In this scenario, even though PPAR*γ* is not required for macrophage differentiation or phagocytic activity, its deficiency is associated with the constitutive onset of an inflammatory milieu, in turn resulting in an enhanced susceptibility to diet-induced obesity, glucose intolerance, and insulin resistance [31]. 

All these findings indicate the crucial role of PPAR*γ* in adipocytes as well as macrophages, although, to date, only two studies [28, 31] have analyzed in-depth the localizations and mechanism of PPAR*γ* recruitment within this cells, trying to address these quite complicated but fundamental questions.

The recent technological advances*—*such as high-throughput sequencing methods and innovative techniques for following the three-dimensional interactions of chromosomes in the nucleus*—*allow to rapidly uncover new layers of complexity within PPAR*γ* world. By using these approaches, it would be of interest to analyze the selective pattern of PPAR*γ* activity within specific cell types, with the final aim to understand how its alterations may affect human health.

Several studies have been performed about *PPARG* gene and its main isoforms, namely, PPAR*γ*1 and -*γ*2, even though other variants have been disclosed [16, 21, 22]. In the near future it would be of great relevance to address also the role of newly described isoforms in physiologic as well as pathologic conditions. 

However, the phenotypic effects described for human PPAR*γ* variants, and various mouse models with altered expression of PPARG mRNA, and often conflicting results from different studies so far performed, unequivocally depict a highly complex picture of PPAR*γ* functions and biology.

## 3. PPARG Target Genes: Polymorphisms, Haplotypes, and Gene Expression

*PPARG* gene nucleotide variations, and their possible phenotype consequences, have been widely and conversely analyzed in the last two decades [7, 23, 71, 72]. Since PPAR*γ* is a transcriptional factor involved in the regulation of several target genes in many tissues, the primary consequence of a genetic variant is likely to be an alteration of expression levels of target genes. 

Although the impact of common single nucleotide polymorphisms (SNPs) in *PPARG* gene on the expression of its target genes is not fully understood, an SNP and/or a combination of them (haplotype) may affect the *PPARG* transcript itself and in turn its ability to regulate gene expression [23].

What does really happen to PPAR*γ* activity in the presence of a DNA polymorphism and/or mutation? Few studies have directly considered the real effect of *PPARG* variants on the *PPARG* expression itself and of its target genes, evaluating the alteration of its binding affinity to PPRE, the promoter efficiency, and other factors that may affect its transactivation ability [73–84]. 

Indeed, most of the studies about nucleotide variations in *PPARG* have mainly focused on the association between a DNA variant and a specific phenotype (such as predictors of diabetes, obesity, and BMI) [57, 85–89] or related biochemical markers (plasma levels of hormones, peptides, or metabolites) demonstrated—or just supposed—to be transcriptionally regulated by PPAR*γ* itself [78, 90–102]. 

The most widely studied SNP in PPARG gene [57, 72, 73, 83], Pro12Ala, occurs in PPAR*γ*2 isoform and has been very often associated to clinical consequences and several alterations of physiological metabolic status [57, 72, 73, 85–87, 89, 103]. About the direct effect of this polymorphism on PPAR*γ* activity, some functional studies have revealed that Pro12Ala confers to PPAR*γ*2 a decreased binding affinity to PPRE and a reduced transactivation ability, both in a luciferase reporter gene assay and in TZD-induced adipogenesis [73, 75].

It has been also shown that in human adipose tissue there were no significant differences in the basal expression levels of some PPAR*γ* target genes (*UCP-2, LPL, p85aPI3K, *and *P* *P* *A* *R* *γ* *1*) between obese Pro12Ala and Pro12Pro carriers, except for a reduction of about 40% observed for *p85aPI3K* gene in the omental fat [78].

To explain the observed discrepancies, between *in vitro* and *in vivo* studies, Kolehmainen et al. speculated that subjects Ala12 homozygous have more relevant differences in gene expression activation compared to Ala12 heterozygous; moreover, it must be considered the interaction of genetic and environmental factors and observed tendency for a higher expression of PPAR*γ*2 in the subcutaneous fat depots of Pro12Ala carriers [78].

In addition, Heikkinen and colleagues (2009) [83] have recently highlighted the importance of metabolic context in modulating Pro12Ala effects, reporting or confirming several associations between this PPARG variant and phenotype traits (Table 1). They have shown that in WAT of Ala/Ala mice some genes were downregulated, whereas a great number of genes were upregulated in muscle. Furthermore, they have interestingly suggested that Pro12Ala might be implicated in G protein function, in sensitization of adiponectin signaling and altered cofactors recruitment [83].

To investigate how Pro12Ala might influence gene expression of molecular targets and in turn the response to exogenous *stimuli*, the functional properties of N-terminal domain should be also considered. In particular, this SNP occurs at position 12 in the N-terminal region of PPAR*γ*2 and shows different transactivation ability than PPAR*γ*1, differing only in its N-terminus. As mentioned above, the additional residues at N-terminus of PPAR*γ*2, encoded by the exon B, confer a trans-activating ability up to tenfold greater than PPAR*γ*1, indicating that *γ*2 isoform is more potent to induce the expression of target genes in the absence of activating ligands [50]. Pro to Ala amino acid change might affect the secondary structure of the protein and consequently its functionality [110]. Indeed, it has been recently shown that proline residues, although counteracting *α*-helix formation, fit well only into N-terminal of *α*-helices, positively modulating the proteins' stability [111].

The direct relationship between PPAR*γ* transcriptional ability and an SNP in the regulatory region of *PPARG* gene, C-2821T, was reported by Muller and colleagues (2003) [79] in the Pima Indians population. This polymorphism, in strong linkage disequilibrium (LD) with Pro12Ala, falls within a putative E2-box in a binding site for *δ*EF1, a transcriptional repressor. Since it has been shown that C-2821T confers to PPAR*γ* an increased transcriptional ability [79], this SNP might be responsible for a decreased binding affinity between *δ*EF1 and E2-box or for a reduced complex stability. Although the mechanism by which these alleles in LD (−2821T and Ala12) function remains uncertain, taken together these findings suggest that Ala12 may alter PPAR*γ*2 transactivation ability, and −2821T may alter transcription of PPAR*γ*2 isoform [79]. Other nucleotide variations, most of them gain- or loss-of-function mutations, have been described in *PPARG* gene. 

A functional study about a rare gain-of-function PPAR*γ*2 mutation, Pro115Gln, highlighted the relevance of phosphorylation at Ser 114 in reducing PPAR*γ* activity; this variation in the ligand independent activation domain of PPAR*γ* affects phosphorylation and renders PPAR*γ* constitutively active, according to increased body mass index (BMI) observed in obese individuals [71, 108]. 

Another *PPARG* nucleotide variation, affecting PPAR*γ* function, occurs in the same domain: a rare frameshift mutation, [A553ΔAAAiT]fs.185[stop186], resulting in a truncated protein in the DBD [76]. Within the same family, this premature stop codon was found in all individuals with insulin resistance and metabolic syndrome (MS), carrying also a similar mutation ([C1984ΔAG]fs.662[stop668]) in PPP1R3A (protein phosphatase1- regulatory subunit 3) [7, 76, 82]. This frameshift is a loss of function mutation that affects heterodimers formation and PPAR*γ* interaction with PPRE in target gene promoters, resulting in a failed transactivation [76]. 

It has been shown, in *vitro, *that four rare mutations in the LBD of PPAR*γ* result in a reduced PPAR*γ* trans-activating ability in the presence of a synthetic ligand, affecting its ability to recruit cofactors, ligands, and RXR*α*: Pro495Leu (also called Pro467Leu), Val318Met (also called Val290Met), Phe388Leu, and Arg425Cys (Table 1) [7, 71, 74, 77, 81, 109]. The first two mutations affect two helices critical for the recruitment of ligand and cofactors and have dominant-negative activity against wild-type PPAR*γ*. The latter, in contrast, are haploinsufficient mutations, occurring in a hydrophobic region that interacts with RXR*α* and ligands [7, 71].

In a more recent study, other rare mutations, occurring in DBD—Cys114Arg, Cys131Tyr, and Cys162Trp—and in LBD −315Stop and Arg357X—of PPAR*γ*, have been described. These variants encode proteins unable to bind DNA, which lack the transactivation ability and show a dominant negative activity consisting in the competitive recruitment of coactivators with wild-type PPAR*γ* (see Table 1) [82].

Furthermore, we recently reported a novel dominant negative mutation in PPAR*γ* LBD, Ser289Cys, associated with colorectal cancer, dyslipidemia, hypertension, and overweight, but no with T2DM. The formation of an S-S bridge, between Cys289 and Cys285, might impede agonist positioning, explaining the demonstrated reduction of transactivation ability of mutant protein [84].

Although some studies have demonstrated the functional impact of *PPARG* nucleotide variations on protein activity and/or stability and on its ability to trans-activate target genes, most of *PPARG* variants have been associated with clinical effects [71, 88, 89] or plasma levels of a protein without investigating *PPARG* expression, isoform abundance, and mRNA levels of target genes. These nonfunctional association studies do not prove—allowing just to hypothesize—the altered expression of *PPARG* target genes. Moreover, it has been demonstrated that gene-gene and gene-environment interactions (i.e., diet, exercise, and age of onset of the disease) may greatly affect the contribution of a specific SNP to the resulting phenotype.

Taken together, these considerations contribute to explain the conflicting results about *PPARG* nucleotide variations obtained in different populations [57, 73, 78, 85–89, 104, 108, 110, 112–115].

For instance, Pro12Ala has been often associated with several diseases and phenotype effects [7, 71, 72], such as increased protection from T2DM onset and insulin resistance, decreased incidence of cardiac disease, higher HDL cholesterol, reduction of BMI in nonobese individuals [57, 73, 85, 87, 103], and increased BMI in obese individuals [86, 91]. A recent study in Russian population supports the association of Pro12Ala with improved insulin sensitivity and the protection from T2DM [89]. Moreover, a recent meta-analysis of 60 association studies also confirms the association between Ala12 allele and reduced T2DM risk [110].

In contrast, two recent conflicting studies in the Indian population have shown that Pro12Ala contributes to T2DM development [105] and do not exhibit any association with MS, T2DM, and obesity, respectively [102]. Gene-environment and gene-gene interactions might strongly contribute to the different Pro12Ala effects observed in the studied populations [23, 116].

This SNP has been also associated to altered plasma levels of LPL, leptin, adiponectin, and resistin. Indeed, it was shown, *in vivo*, that Ala12 allele is associated with a reduced LPL activity in postheparin plasma [93]; higher leptin levels were observed in Pro12Ala compared to Pro12Pro carrier women [92]. The effect of Pro12Ala on increased leptin levels is likely to be supported by a study in women with functional hyperandrogenism (FOH), in which the authors demonstrated that Ala allele was more frequent in FOH women than in healthy controls (36% versus 28%) and that leptin levels were higher in nonobese FOH women compared to controls [100].

Also the association between Pro12Ala and adiponectin plasma levels seems controversial: in the Japanese population Ala12 allele is associated with reduced serum adiponectin levels [94, 96] whereas no significant effect of this polymorphism on serum adiponectin was observed in polycystic ovary syndrome, healthy women, and in Asian Indians [98, 101].

In a study by Wang et al. (2004) in [97], it has been reported that Ala12 allele might affect the expression of a gene *RETN *encoding another adipose tissue-related molecule, the resistin, in the Chinese population; both heterozygous and homozygous Ala12 carriers showed lower plasma resistin levels compared to homozygous Pro12 carriers [97]. On the opposite, a recent report in an Indian population described no statistically significant differences in resistin plasma levels between Pro12 and Ala12 carriers (both heterozygous and homozygous) [102].

Pro12Ala has been described in linkage disequilibrium (LD) with another common *PPARG* variant, C1431T; this silent SNP, occurring in the exon 6, is also known as His477His and C161T of exon 6 [71]. It has been observed that when Pro12Ala is in LD with C1431T SNP, its protective effect on T2DM development disappears [87], while the consequences on BMI are potentiated [106]. 

The lack of functional findings within the above-described association studies and possible influence of ethnicity, environmental and genetic factors are likely to explain the controversial results so far reported. Moreover, due to LD between polymorphisms, determining the relative contribution of each SNP on the resulting phenotype is quite difficult.

For instance, different studies report that 1431T allele is associated with an increased BMI in obese Finns [91], a reduced risk of diabetes in a large Asian population [107], and not at all associated with T2DM, obesity, and BMI alteration [88]. 

About its effects on plasma proteins levels, C1431T has been associated with increased leptin levels [90]. Also Valve et al. [91] observed higher leptin levels in the obese women with C1431T than other obese women studied; this polymorphism was associated with increased fat mass, and, albeit in this study, the authors hypothesize that higher leptin levels were entirely due to increased adipose tissue mass and not directly linked to PPAR*γ*-dependent transcriptional regulation [91].

Moreover, also resistin levels were significantly increased in individuals carrying C1431T whereas the Pro-C haplotype was more frequent in groups with lower resistin levels. In contrast, Pro-T and Ala-T haplotypes showed increased frequency in groups with higher resistin levels although statistically not significant [102]. 

Moreover, nucleotide variations in putative regulatory regions of PPARG have been associated, with different extents, to human diseases. Indeed, we recently identified A-2819G SNP in PPARG promoter and observed a significant association with T2DM and proliferative retinopathy in diabetic females whereas no linkage disequilibrium with Pro12Ala nor association with obesity was observed [88]. It has been hypothesized that this SNP might alter *PPARG* transcript abundance influencing in turn the expression levels of some PPAR*γ* targets involved in the eye physiology [88]. 

Other three variants in PPARG putative promoter have been identified: A-14G, C-681G, and C-689T [71], even though their impact on PPARG transcription and function has not been completely elucidated. These polymorphisms may possibly affect the expression of some PPAR*γ* molecular targets, since C-681G and C-689T were associated with increased plasma LDL levels and A-14G with a decreased activity of PPAR*γ*4 promoter [80, 95, 99].

The introduction of massively parallel sequencing platforms, which have offered to researchers the possibility to identify, in a single experiment, point mutations and/or gross genomic rearrangements, within coding and yet unexplored regulatory regions of disease-causing genes, will surely represent a powerful tool to systematically discover variations in *PPARG *gene, possibly giving a causal link to human diseases. 

## 4. Next-Generation Sequencing Technologies and Transcription Factors: ChIP-Seq, Targeted Resequencing, and RNA-Seq

Any genetic information is conveyed from DNA to proteins via mRNA, through a complex and finely regulated process. Unraveling how these genomic information are then translated into gene regulation has been for many decades an intriguing field, fulfilled by many advances, speculations, and scientific debate. To achieve this tuned regulation, the concerted action of multiple *cis*-acting proteins, able to specifically bind *cis*-regulatory elements, such as promoters and enhancers, is needed [2, 117]. Moreover, since the basal transcriptional activity, resulting from the binding of so-called general TFs to the *core* promoter, is usually low, different site-specific TFs participate to the recruitment and/or the stabilization of general TFs' complexes, increasing the cell transcriptional rate. Moreover, histone-modifying enzymes may be recruited by other factors—binding to distal enhancer regions—and determine a favourable chromatin environment and a subsequent transcriptional enhancement. On the other hand, the transcription can be negatively modulated through the binding of repressive factors to distal silencer regions or the competition with TFs themselves.

To understand PPAR*γ*-mediated phenomena in a specific cell/tissue/organ one cannot ignore the consideration that PPAR*γ* is a transcription factor. Its mechanistic understanding represents a prerequisite for fine-tuning the therapeutic activities of *PPARG*. 

More generally, several human diseases have been directly linked to alterations in the gene expression caused by defects in the structure and/or function of a key transcriptional regulator [2] although it is arguable that many other “TF-disease associations” still remain to be identified. Expanding our understanding of how site-specific TFs contribute to gene expression regulation, and in turn how alterations in both TF structure and activity may account for a specific disease phenotype, appears to be a crucial endpoint. 

In this context, the specific case of PPAR*γ* is not an exception, rather it is likely to be one of the most representative candidate genes in “TF-disease” association studies, for its involvement in many physiological and also pathological processes [7].

To this aim, the introduction of massively parallel sequencing platforms in the 2004, coupled with the recent advances in chromatin immune-precipitation (ChIP) followed by sequencing (ChIP-seq), has clearly revolutionized the way we approach to—and also study—different biological phenomena [118–121]. Although all the sequencing platforms commercially available use different sequencing chemistry and methodological procedures, also varying in the number of sequenced reads, read length and error characteristics, they all are based on the generation of libraries to sequence, and the miniaturization of individual sequencing reactions [121]. Unlike previously used tag-based sequencing methods, such as Serial and Cap Analysis of Gene Expression (SAGE and CAGE, resp.), Polony Multiplex Analysis of Gene Expression (PMAGE), NGS libraries do not require a prior step of cloning before sequencing. Moreover, a common feature of NGS platforms is the template binding to a solid surface or support (immobilization by primer or template) or its indirect immobilization (by linking a polymerase to the support) [122]. However, whatever are the sequencing chemistry and the methodological procedures used, a single NGS platform can generate a large amount of data up to 2 gigabases (Gb) of sequence reads per day, shifting the effort of researchers from biology to bioinformatics.

These platforms have been quickly applied to many scientific contexts, giving rise to many “Seq” protocols, specifically developed and suited for a particular research branch, from transcriptomics (RNA-Seq) to the targeted resequencing for the identification of disease-causing nucleotide variations (CNV-Seq and DNA-Seq), including DNA-protein interaction studies (ChIP-Seq) and genome-wide profiling of epigenetic marks (Methyl-Seq). 

Although it is beyond any doubt that NGS platforms have changed the way we think about many scientific issues, one of the broadest and useful applications of this technology is towards the identification of the genetic causes of inherited diseases, both mendelian and multifactorial. 

**Figure 2:**
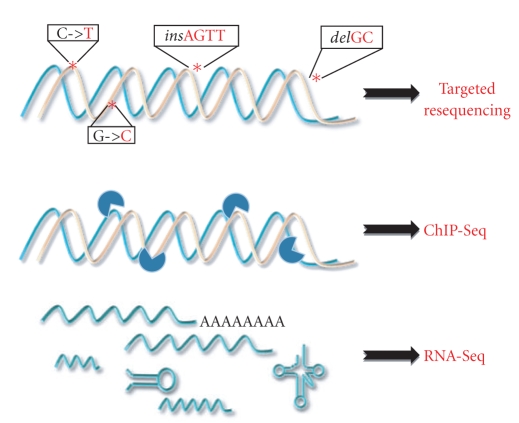
Innovative approaches by using next generation sequencing technologies. Next generation sequencing can be applied to many scientific contexts: targeted resequencing for the identification of disease-causing nucleotide variations for both coding and unexplored regulatory regions of genes (CNV-Seq and DNA-Seq); ChIP-Seq, for DNA-protein interaction studies coupling chromatin immuno-precipitation (ChIP) and massively parallel sequencing; RNA-Seq, for whole transcriptome studies, including expression levels of known and yet unknown transcripts (both coding and non-coding), differential splicing, allele-specific expression, RNA editing, and fusion transcripts (see review [121]).

In light of this, deeply investigating *PPARG*—from DNA variations to gene expression and its regulation—will surely enhance our understanding about its involvement in health and disease. Identifying novel nucleotide variations, both point mutations and gross genomic rearrangements, within coding regions and yet unexplored intronic and regulatory regions of *PPARG* by targeted resequencing (on NGS platforms) will be the first brick towards building a more complete and detailed view of *PPARG* function and activity (summarized in Figure 2). In addition, the possibility to identify the exact position of its binding sites and thus draw a complete high-resolution binding map across the genome (by ChIP-Seq) [123, 124], combined with the large amount of useful whole transcriptome data obtained by RNA-seq, will provide an unprecedented level of accuracy and complexity than ever done (see Figure 1) [125]. 

### 4.1. NGS for the Targeted Resequencing

The whole genome resequencing of affected individuals' genomes by the use of NGS platforms is likely to represent the most powerful approach to identify single nucleotide variants and/or genomic rearrangements (insertions, deletions, and copy number variations) within disease-causing genes. Nonetheless, it is clear that such genome-wide approach cannot be used for a routine mutational screening in wide number of affected individuals, due to the high computational and economic effort required, particularly considering that there are few research groups in big companies and/or large corporations, as well as big public and private world leading research institutions, able to sustain these costs. 

Thus, targeted resequencing of a small number of candidate genes or disease *loci* appears to be the only reliable way to obtain the high accuracy of NGS data at the accessible costs of a standard array analysis. On the other hand, it appears crucial to have efficient and cost-effective capture methods to enrich the sample with “high-value” genomic regions to sequence in order to avoid off-target sequencing. 

In light of this, different techniques have been recently developed allowing researchers to enrich their sample of target genomic regions to be further sequenced. Multiplex PCR amplification of specific target regions was first used for candidate gene approaches, to enrich the samples with regions of interest, further processed to prepare libraries prior to sequencing [126–128]. Another approach is the capture-by-hybridization [129]. Efficient array-based capture approaches (custom *in-situ* synthesized oligonucleotide microarrays) have been successfully used to enhance the sequencing template enrichment [130–133]. Companies, such as NimbleGen, have recently developed microarrays for the capture-by-hybridization of thousands of predefined genomic regions, mainly coding regions (exons), widely used for targeted resequencing experiments [134]. Several research groups have clearly shown that the above-described capture methods are playing a crucial role in driving targeted resequencing applications of NGS platforms [129].

Since most of human genetics studies have so far mainly focused on protein-coding exons, these regions usually represent high-value targets for targeted resequencing, even though this approach can be—and we believe in most cases it must be—extended to gene regulatory regions (upstream the translation start sites and introns). Indeed, the identification of nucleotide variations in putative or already known regulatory sequences, within non-coding genomic regions, is therefore of great relevance for future research. This approach appears to be very promising above all for the study of TFs binding sites, since their involvement in human disease, both mendelian and multifactorial [135]. Barrio and colleagues (2009) [135] first identified, by target resequencing of a genomic region encompassing about 20kb, non-coding variations associated with two kinds of red cell aplasia, demonstrating that non-coding *RPS19* gene sequence variants contribute to the high clinical variability observed in red cell aplasia. They hypothesized that specific alleles in these non-coding regions may alter the binding of regulatory proteins and/or TFs, possibly altering or removing an important stimulus for hematopoiesis [135].

In this context, the possibility to have high enrichment for the both coding and unexplored regulatory regions of *PPARG, *coupled to the targeted resequencing on NGS platforms, will represent a very powerful approach for researchers. Indeed, it is likely to allow the identification of all the potential risk-conferring variations, within its coding regions, of putative novel single nucleotide variations (mutations and SNPs) and insertions/deletions or other genomic rearrangements, possibly associated to human diseases. It will also allow gaining further insights into the genomic architecture of its regulatory regions, offering the possibility to rapidly and accurately identify potential sources of variation responsible for the alteration of its mRNA levels. Moreover, the specific enrichment of target regions, followed by targeted resequencing, could also be performed on well-known PPAR*γ*-regulated genes in specific pathways. 

Indeed, since several studies performed on PPAR*γ* target genes have not unequivocally shown a clear correlation between SNPs and the related human diseases, by using these approaches it will be easier to identify specific alleles in non-coding regions of target genes and verify whether these nucleotide variations are responsible for the alteration of known PPRE—and in turn of PPAR*γ* binding to these elements—finding a direct and functional link to the disease.

### 4.2. Transcription Factors and ChIP-Seq

Thanks to the introduction of NGS platforms, widely used approaches of chromatin immuno-precipitation followed by microarray (ChIP-chip) have been flanked—and in many cases substituted—by ChIP-seq protocols. Indeed, in ChIP-seq, the DNA fragments of interest (i.e., binding sites for a TF) are directly sequenced instead of being hybridized on a chip-array. Thanks to the high resolution, coverage, the wider dynamic range, and the absence of hybridization-based artifacts, ChIP-Seq allows now researchers to improve both quantity and quality of produced data. Moreover, fundamental advances toward a more accurate definition of the consensus sequences for the binding of TFs have been done [136].

To date, this novel approach, which couples in a single experiment a standard ChIP assay to the large-scale massive sequencing of target genome regions, allows researchers to obtain a more complete map of TFs-DNA interactions [2]. Drawing a precise map of TFs binding sites, core transcriptional machinery, and other DNA-binding proteins is a crucial step towards the identification of gene regulatory networks underlying physiological as well as pathological processes [136]. 

In particular, since PPAR*γ* acts in combination with RXR as heterodimer and requires the cooperation with many different tissue-selective factors, understanding the differential spatial and temporal recruitment of PPAR*γ*:RXR complex to target genes is likely to improve our knowledge about PPAR*γ* biology.

In a recent study, Nielsen et al. [28] by ChIP-Seq on NGS platform (Illumina, Roche) obtained a PPAR*γ*- and RXR-binding sites map during the adipocytes differentiation of 3T3-L1 cells [28]. In particular, they sequenced a total of about 86 million of fragments (divided for the six days of the analysis on the adipocytes) derived from PPAR*γ* ChIP assay and about 50 million derived from RXR ChIP. They demonstrated that spatial and temporal recruitment of PPAR*γ* and RXR to target genes varied during adipogenesis (Figure 1). More in detail, they observed that in the very early stages of adipocyte differentiation, coinciding with the very low levels of PPAR*γ* at day 0, only nine PPAR*γ* target sites were detectable, and however this number remained low at day 1. In contrast, a high DNA occupancy by RXR alone was detected. More interestingly, going on with the differentiation process, most of these sites become occupied by PPAR*δ*:RXR complexes. A subsequent switch—starting at day 2—between PPAR*δ* and PPAR*γ*, which becomes the main RXR partner throughout the adipogenesis, coincided with a significant increase in both PPAR*γ*1 and PPAR*γ*2 expression [28–30]. They identified >5000 high-confidence PPAR*γ*:RXR-binding sites in adipocytes coinciding to the majority of induced genes. *In silico* analysis allowed to observe that binding occurs in the proximity of genes involved in lipid and glucose metabolism. The highest number (about 7000) of PPAR*γ*:RXR-binding sites was observed at day 6. This genome-wide ChIP-Seq analysis allowed to confirm the binding of PPAR*γ*:RXR heterodimer to well-established PPREs in already known target genes. In addition, novel target sites in introns of different genes were also identified.

ChIP-Seq was also recently used by Lefterova and colleagues (2010) [31] to address a critical issue affecting several reports about PPAR*γ* function, the specificity of action, that is, how PPAR*γ* modulates target genes in some cell types but not others. By using this innovative approach, the authors determined which cell-type-specific cofactors are recruited by PPAR*γ* in mouse macrophages and adipocytes [31]. Indeed, it has been widely demonstrated the PPAR*γ* transactivation ability on target genes, with characteristic cell-type specific patterns of gene modulation, but the molecular basis of such a specificity has not yet been fully understood. 

As generally assumed for other TFs, it has been postulated that its cell-type specificity might be due to a differential binding to consensus sequences in the regulatory regions of target genes or a differential ability to recruit chromatin remodelling enzymes [38]. The authors identified a specific molecular signature of PPAR*γ* binding, by massively sequencing—overall the mouse genome—the regions directly bound by PPAR*γ*. This analysis revealed that PPAR*γ* cooperates with some cell-type-specific factors, PU.1 and C/EBP*β*, in the defining the specificity of action for PPAR*γ* in each cell type (macrophages and adipocytes, resp.) (Figure 1). PPAR*γ* in macrophages binds uniquely at genomic sites located in the proximity of immunity-related genes and specifically colocalizes with PU.1 in areas of open chromatin and in presence of histone acetylation whereas, in preadipocytes, the presence of a repressive histone signature excludes PPAR*γ* from macrophage-specific sites. In this case it has been shown that PPAR*γ* is able to open the chromatin and increase histone acetylation at adipocyte-specific genomic sites. This paper demonstrates that, at least in these cell types, the transcriptional regulation exerted by PPAR*γ* is attributed to a differential recruitment of specific cofactors functioning as scaffolds for chromatin remodelling enzymes.

Above described works have clearly shown the great potential of sequencing-based ChIP assays, which do not require *a priori* information about the genomic position of TFs binding sites and allow to generate high-resolution binding maps in response to a specific *stimulus* [123, 124]. However, as demonstrated in a recent work by Reddy and colleagues (2009) [125], coupling ChIP-Seq to RNA-Seq (described in detail in the next paragraph) for studying the response of a TF to a specific drug allows to examine well-known models at much greater depth and detail. In particular, they obtained a comprehensive map of glucocorticoid receptor binding to DNA overall the genome by ChIP-Seq, and measured related changes in gene expression by RNA-Seq, in response to treatment with dexamethasone [125].

We firmly believe that combining a sequencing-based ChIP assay to high-throughput transcriptome analysis by RNA-Seq on NGS platforms, above all for inducible transcription factors (and PPAR*γ* among them), will surely provide a complete, accurate, and reliable source of useful of data, enabling to complete, piece by piece, the intricate puzzle of PPAR*γ* functions.

### 4.3. Discovering the Transcriptional Landscape through RNA-Seq

Since the end of the 90s the term “transcriptome” was used to describe the identity of each expressed gene in a specific cell type and/or tissue/organ/organism, and of its related transcriptional levels [137]. It was first believed to consist of 80–90% of ribosomal RNA (rRNA), 5–15% of transfer RNA (tRNA), and the remaining fraction of messenger RNA (mRNA), with most of the genome consisting of untranscribed and genetically inert regions.

In contrast, recent evidences have shown that both intragenic and intergenic sequences cannot be any longer considered as “junk DNA”, but they represent one of the main driving force accounting for diversity and biological complexity of all living organisms [121]. Indeed, several studies have demonstrated an unexpected level of complexity of the eukaryotic transcription, showing its pervasive nature with almost the full length of nonrepeat regions of the genome being transcribed [138, 139]. 

Hence, interpreting the complexity of a whole transcriptome is likely to be a crucial endpoint for unraveling the role of functional elements of a genome, and, in light of this, the introduction of NGS platforms has provided researcher a powerful tool for analysis in a single experiment. 

Indeed, the rapid diffusion of RNA-Seq protocols has raised the possibility to quantify the differential expression of transcripts in both physio- and pathological conditions and to identify and characterize all the transcripts (both protein-coding and non-coding) expressed within a specific cell and/or tissue—at a particular development stage or after an endogenous or exogenous *stimulus*—correctly determining the splicing and the structure of genes. Unlike hybridization-based gene expression methods (microarray) and tag-based sequencing (i.e., CAGE and SAGE), RNA-Seq does not require prior knowledge of any gene sequence (as occurs for microarrays) or laborious and time-consuming steps for the cloning and sequencing (as occurs for existing tag-based approaches) (reviewed in [121]). 

Several recent studies have clearly demonstrated the advantages of using RNA-Seq in the interrogation of transcriptomes under multiple conditions, such as cell proliferation, differentiation, and various environmental stress [140–148].

In this context, due to the crucial role of PPAR*γ* as TF involved in many cellular pathways, investigating the PPAR*γ*-dependent regulation of target genes expression via RNA-Seq in a single experiment represents a great challenge. 

Whereas previously described ChIP-Seq allows to draw a binding map of PPAR*γ* to PPRE, activating or repressing target gene expression, directly identifying (by RNA-Seq) the gene expression response to PPAR*γ*-modulating drugs (agonists such as TZD), or in particular development conditions (during adipogenesis), will provide researchers the opportunity to directly measure its ability to modulate the transcription of specific genes in a cell/tissue specific manner. 

Since high-throughput sequencing has definitely proved to be a powerful and quantitative method to sample the transcriptomes at single nucleotide resolution [149], the use of RNA-Seq is likely to shed a new light on the specificity of action of PPAR*γ* in different cell types or tissue, in both physiological and pathological conditions. Several unsolved questions about the “real” impact of PPAR*γ* on the regulation of target gene expression—in several conditions—can now be fruitfully addressed by the use of NGS.

## 5. Concluding Remarks

Over the past years, PPARs, and especially PPAR*γ*, have emerged as crucial transcription factors modulating the expression of genes involved in several important pathways and biological processes and, noteworthy, in human diseases. Despite the huge knowledge in the field, future research efforts will undoubtedly reveal novel mechanisms through which PPAR*γ* integrates these complex physiological and pathological pathways. Particular attention should be given to the question of how the selective effects of PPAR*γ* are achieved in different cell types. It will also be of great importance to understand the subtle mechanisms dictating this selectivity of action through the study of its different isoforms, genetic variations, and their recruited cofactors able to remodel the chromatin structure. Knowing all the PPAR*γ* targets is a prerequisite for a full understanding of the metabolic defects that occur in people with *PPARG* mutations and/or variation and will help in the interpretation of effects—and also side effects—that can occur with PPAR*γ* agonists already in clinical use. Thus, to have a complete picture of PPAR*γ* functions and implications, studying altogether these aspects, through the use of massively parallel sequencing platforms, will provide a way to better characterize the actions of *PPARG* products and agonists.

## Figures and Tables

**Table 1:** Nucleotide variations within coding and regulatory regions of PPARG.

Variant	Disease/trait	Outcome/Association	References
Pro12Ala	T2DM Insulin resistance	Conflicting results about association to T2DM and insulin resistance. When in LD with C1431T no protection from T2DM development	[57, 73, 83, 85, 87, 89, 102, 104, 105]
	Cardiac disease	Decreased incidence of cardiac disease	[103]
	HDL	Higher HDL cholesterol	[73]
	BMI	Reduction of BMI and fat and lean mass in nonobese (potentiated when in LD with C1431T) and BMI increase in obese individuals	[73, 83, 86, 91, 106]
	LPL	Reduced LPL activity and levels.	[83, 93]
	Leptin	Increased leptin levels	[92, 100]
	Adiponectin	Reduced adiponectin levels	[94, 96, 98, 101]
	Resistin	Reduced resistin levels	[97, 102]
	Bone features	Increase of total bone area and bone mineral content in Ala/Ala mice.	[83]

C1431T	BMI T2DM Leptin Resistin	Increased BMI and fat mass. Reduced risk of T2DM. Increased leptin levels. Increased resistin levels.	[88, 90, 91, 102, 107]

Pro115Gln	BMI	Increased BMI in obese individuals	[108]

[A553ΔAAAiT]	Insulin resistance T2DM Hypertension	In association to 662stop668 mutation in *PPP1R3A* is responsible of variable hyperinsulinemia, T2DM, hyperlipidemia, hypertension, and dyslipidemia.	[76]

Pro495Leu and Val318Met	Insulin resistance T2DM Blood pressure Partial lipodystrophy Protein plasma levels	Severe insulin resistance, TD2M, and early-onset hypertension. Dyslipidemia, preservation of abdominal fat with selective loss of gluteal and limb subcutaneous fat; inability to trap and store NEFA in the postprandial state, hepatic steatosis; reduced adiponectin plasma levels.	[74, 76]

Phe388Leu	Partial lipodystrophy and related features.	Lipodystrophy and dyslipidemia less severe, with absence of fat depots on the upper arms, phlebectasia of limb veins and of hepatic steatosis. Atherosclerosis, polycystic ovarian disease, increased C-peptide concentration, higher insulin resistance.	[77]

Arg425Cys	Partial lipodystrophy T2DM	Diabetes mellitus and hypertriglyceridemia previous to the development of limb and facial lipoatrophy; loss of subcutaneous fat, except for sc truncal fat. Hirsutism in a female carrier.	[109]

Cys114Arg Cys131Tyr Cys162Trp 315Stop Arg357X	Partial lipodystrophy and related features.	Reduced body fat, partial lipodystrophy of limb and gluteal depots, insulin resistance, hepatic steatosis, severe dyslipidemia, increased triglycerides levels, low HDL levels. Not for all: early-onset hypertension, cutaneous eruptive Xanthomata, pancreatitis.	[82]

Ser289Cys	Colorectal cancer	Colonic lesions, reduced restraint of cell proliferation both in vitro and in vivo, interference with the inflammatory pathway in tumor tissues and proximal normal mucosa	[84]

A-2819G	T2DM and diabetic retinopathy	Association with T2DM and proliferative retinopathy in diabetic females.	[88]

C-689T C-681G	BMI LDL	Increased BMI. Increased LDL levels.	[95, 99]

A-14G	Partial lipodystrophy MS	−14G associated with familial partial lipodystrophy subtype 3 (FPLD3). It has been found MS and a relative reduction of gluteal and extremities'fat.	[80]
